# 

**Published:** 1886-12

**Authors:** 


					﻿DECEMBER, 1886
1,IAI l>
Jo\kwi -
HEAII H
’ ESTABLISHED 18^4
U°±- 33
o/lfe 12
OFFICE; 208 BROADWAY, EVENING POST BUILDING, Eoom 11. N. Y.
Entered as Second-class Matter at the Post-Office, New York.
				

